# The Effect of a Tailored Educational Flyer on Colorectal Cancer Screening Among Rural Residents: Lessons Learned from a Pilot Randomized Trial

**DOI:** 10.3390/cancers16213645

**Published:** 2024-10-29

**Authors:** Jungyoon Kim, Cheryl Beseler, Melissa Leypoldt, Roma Subramanian, Tamara Robinson, Karen Funkenbusch, Jason Foster, Susan Harris, Aaron Yoder, Emma Hymel, Shinobu Watanabe-Galloway

**Affiliations:** 1 Department of Health Services Research & Administration, College of Public Health, University of Nebraska Medical Center, Omaha, NE 68198, USA; 2 Department of Environmental, Agricultural & Occupational Health, College of Public Health, University of Nebraska Medical Center, Omaha, NE 68198, USA; chbeseler@unmc.edu (C.B.); aaron.yoder@unmc.edu (A.Y.); 3 Nebraska Department of Health and Human Services, Lincoln, NE 68508, USA; melissa.leypoldt@nebraska.gov; 4 College of Communication, Fine Arts and Media, University of Nebraska Omaha, Omaha, NE 68182, USA; rsubramanian@unomaha.edu; 5 Nebraska Cancer Coalition, Lincoln, NE 68521, USA; 6 University of Missouri Extension, Columbia, MO 65211, USA; funkenbuschk@missouri.edu; 7 Department of Surgery, College of Medicine, University of Nebraska Medical Center, Omaha, NE 68198, USA; jfosterm@unmc.edu; 8 University of Nebraska—Lincoln, Extension, Lincoln, NE 68583, USA; susan.harris2023@gmail.com; 9 Department of Epidemiology, College of Public Health, University of Nebraska Medical Center, Omaha, NE 68198, USA; ehymel@unmc.edu (E.H.); swatanabe@unmc.edu (S.W.-G.)

**Keywords:** colorectal cancer, FIT, screening, intervention, rural, education, small media

## Abstract

**Background/Objectives**: Stool-based tests, such as the fecal immunochemical test (FIT), have been widely used for increasing colorectal cancer (CRC) screening. Small media, such as printed materials or flyers, are known to be an effective intervention to increase CRC screening by fecal tests. However, more evidence is needed to determine whether such small media are effective in improving screening uptake of a mailed FIT intervention targeted at rural populations in the USA. **Methods**: In this randomized study, 1230 FIT kits were mailed from July to December 2022 to rural Nebraskans aged 45–74 who were not up to date on CRC screening. Half of the participants (n = 608) also received a tailored, one-page, gender-specific educational flyer created based on focus groups with rural residents. Logistic regression was used to determine predictors of returning the FIT. **Results**: Study participants were predominantly female (76%), non-Hispanic White (83%), and within the age group of 55–64 (43%). Overall, 192 (15.6%) kits were returned (16.1% from the flyer group; 15.1% from the no-flyer group). However, we found no significant differences between the flyer and no-flyer groups (Adjusted Odds Ratio [AOR]: 1.21; 95% CI: 0.88–1.66). Females (AOR: 1.78; 95% CI: 1.19–6.14) and the oldest (65–74) age group (AOR: 5.03; 95% CI: 2.78–8.47) were more likely to return FIT kits than males and the youngest (45–54) age group. **Conclusions**: A tailored educational flyer was not effective in improving the CRC-screening-rate-by-mailed-FIT approach for rural populations. Future research should explore the content, timing, and mode of delivery of educational interventions as well as other multi-component strategies to improve screening rates. Public health officials might also consider developing strategies targeted at males and younger (45–54) age groups.

## Simple Summary

Despite the benefits of stool-based tests for colorectal cancer screening, the return rate is low. In this study, we tested if adding a tailored, one-page educational flyer to the mailed stool kits targeted at rural Midwestern residents in the USA improved the kit return rates. Overall, there was no significant difference in return rate between the groups that did and did not receive the flyer. However, females and older participants were more likely to return the kits compared to males and younger participants. To improve the kit return rates of male and younger participants, future programs should consider how the content and design of the flyer can be more closely tailored to the audience’s needs and preferences as well as how the flyer can be combined with other approaches (e.g., reminders).

## 1. Introduction

It is estimated that 153,020 people will be diagnosed with colorectal cancer (CRC), and 52,550 individuals will die from CRC in the USA in 2023 [1]. From 2015 to 2019, the age-adjusted incidence rate of CRC in Nebraska was higher than the US rate (41.9 vs. 37.7 per 100,000) [2]. Additionally, the age-adjusted mortality rate of CRC was higher in Nebraska compared to the overall US rate (14.3 vs. 13.1 per 100,000) [2]. The incidence of CRC is higher among rural residents compared to urban residents in the USA (43.9 vs. 40.1 per 100,000), due in part to a higher prevalence of risk factors, including obesity and limited healthcare access [3,4]. 

Currently, the United States Preventive Services Task Force (USPSTF) recommends all adults begin screening for CRC at the age of 45 for early detection and prevention of CRC [5]. Despite these recommendations, only 67.1% of adults in the USA are up to date on CRC screenings [6]. In a study of primary clinics in three states in the Midwest, it was found that rural residents had 4–9% lower odds of receiving CRC screening than urban residents [7]. Furthermore, a nationwide study using Surveillance, Epidemiology, and End Results Program data reported that patients living in rural areas were more likely to be diagnosed with CRC at later stages than those living in urban areas [3]. In Nebraska, it has been observed that rural primary care patients are less likely to be up to date on CRC screening than their urban counterparts (74.4% vs. 88.1%), and colonoscopy use in the past 10 years has been significantly lower among rural patients than urban patients (71.9% vs. 87.5%) [8].

Barriers to CRC screening in rural areas include a lack of prevention attitudes toward cancer, perceived lack of privacy, shortage of specialists, and distance to test facilities [9]. In a survey-based study of Nebraska residents, individuals living in rural areas were more likely to report cost as a barrier to CRC screening and were less likely to report that CRC can be prevented compared to those living in urban areas [8]. Furthermore, focus groups with rural residents in Nebraska also identified a lack of knowledge of screening guidelines and screening tests as barriers to screening [10].

Fecal immunochemical tests (FIT), which screen for CRC by detecting blood in stool, are recommended by the USPSTF to be completed annually for individuals with an average risk for CRC [6]. FIT tests can be completed at home and require only one stool sample, making them more popular than other CRC screening methods [11]. Previous research has indicated that stool tests are effective for increasing CRC screening uptake among rural residents, in part by increasing community access to screening through the direct mailing of kits [12]. In addition to reducing travel time to healthcare facilities, FIT tests may also help to address concerns about CRC screening among rural residents, including fear or pain associated with colonoscopies and embarrassment [13]. In a study of veterans in Iowa primarily living in rural areas, 89% reported that FITs were easy to use and convenient [14]. Previous studies in rural settings have also highlighted the importance of incorporating tailored education in CRC screening interventions to improve screening rates [12,15,16].

The Nebraska Department of Health and Human Services (DHHS) partnered with the University of Nebraska Medical Center College of Public Health to increase CRC screening in rural areas of the state. The DHHS identified regions of high-need populations in Nebraska to target for the distribution of FIT tests in combination with a tailored educational flyer to increase uptake rates. The objective of this study is to determine whether individuals who received an educational flyer were more likely to return their FIT kits, compared to individuals who did not receive an educational flyer.

## 2. Materials and Methods

This study was conducted in accordance with the CONSORT guidelines [17]. This study did not require Institutional Review Board approval as it was designated as a public health project as defined by 45 CFR 46.104(d)(5).

### 2.1. Participants

Participants were selected from existing DHHS datasets. All were enrolled or screened in the last two years in one of the DHHS preventive screening programs. Two separate datasets were used: the Nebraska Colon Cancer Registry (NCCR) and Nebraska’s breast and cervical cancer screening program known as the Every Woman Matters Program (EWMP). Participants were men and women aged 45–74 who had never been screened or had not been screened with a home stool kit in the last 10 months. Individuals screened with a colonoscopy within the last 9 years were excluded. Three geographic areas were chosen based on the state cancer registry data; all were rural, with two having the highest colorectal cancer incidence (Zone 12) mortality rates (Zone 13) in the state and the third (Zone 1) having the lowest screening rate in the state.

### 2.2. Intervention

**Figure 1:**
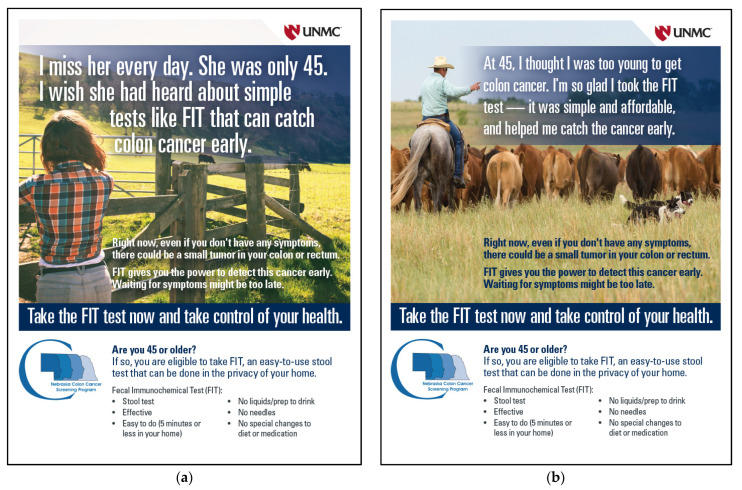
Educational flyers tailored toward rural residents: (**a**) a flyer image for female participants; (**b**) a flyer image for male participants.

The intervention was a one-page educational flyer tailored toward rural residents. Two separate flyers were created based on feedback from rural men and women, who participated in a focus group study [18]. The questions in the focus group guide were informed by factors that are known to influence CRC screening use, especially among rural residents [9], and by using the conceptual framework by Christy and Rawl [19]. We used the concept mapping approach to engage participants and identify statistically the priority responses of barriers and facilitators of CRC screening for men and women [20]. This study generated a list of promoters and motivators for CRC screening, and the findings clearly suggested some gender differences. For example, for rural men, we tailored a message to highlight age eligibility, affordable cost, and easiness of taking the FIT test. For rural women, we highlighted the benefits of early detection, the easiness of taking the test, and age eligibility (Figure 1). 

### 2.3. Study Design

This was a randomized, parallel-group study to examine the return of the stool kit for CRC screening from July to December 2022. We used the stratified randomization approach. The list of participants was divided in half by dataset (NCCR and EWMP), followed by geographic area (Zones 1, 12, 13), and finally by gender (male and female) to create six lists. The potential participants’ names in each of the six lists were alphabetized, and the lists were divided in half, with half receiving the educational flyer and the other half receiving standard information with the kit. The participants were blinded to the intervention. All participants received a reminder letter from a physician.

### 2.4. Measures

Demographic variables included gender (male, female), race (Asian, Black, Mexican, Native American, Pacific Islander, White, unknown), Hispanic ethnicity (yes or no), and age. Race and ethnicity were further categorized into non-Hispanic White, Hispanic, and non-Hispanic Other due to small numbers in some categories. Age was categorized into three age groups: 45–54, 55–64, and 65–74. The inclusion of educational materials in the FIT kit distribution was coded as yes = 1 and no = 0. Variables were created for the three geographic zones (Zones 1, 12, 13) that participants were selected from and two sources of contact (NCCR and EWMP). A variable was created to represent the return of the FIT kit (returned = 1, not returned = 0).

### 2.5. Statistical Analysis

We first assessed the effectiveness of the randomization of intervention group assignment by testing for significant differences in gender, age, race/ethnicity, recruitment source, and recruitment region using a chi-square test for independence for categorical variables and a two-sample t-test for age. Descriptive statistics were calculated for each sample of participants to describe their gender, age, race/ethnicity, recruitment source, and recruitment region. Differences between those who returned the FIT kits and those who did not were compared using the chi-square statistic on the demographic characteristics. Logistic regression analysis was used to calculate the odds of returning the FIT kit between the flyer group and the no-flyer group. Odds ratios and 95% confidence intervals were calculated to estimate the effect size for those who returned the FIT kit.

## 3. Results

### 3.1. Sample

A total of 1230 FIT kits were sent out to those who were not currently being screened for CRC. The sample was primarily female (n = 939, 76.3%) and White (n = 1001, 83.6%). Hispanics comprised 13.3% of the sample (n = 159). The mean age of the sample was 60.2 (SD = 7.60, range 45–74). Most participants were recruited from Zone 1 (68.7%), and 60.0% were recruited from the coalition database (NCCR).

As shown in Table 1, flyers were included in 608 of the 1230 kits mailed out (49.4%). The characteristics of those receiving the informational flyer did not differ statistically from those who did not receive the flyer by gender, race, ethnicity, recruitment source, or recruitment region (*p*-values 0.49–0.79). The group receiving the flyer was slightly younger (mean = 59.6, SD = 7.90) than those not receiving the flyer (mean = 60.8, SD = 7.24) (*p* = 0.01). These differences in age between intervention and control groups are not likely to be large enough to create a bias. Significance is probably a result of a large sample size. After categorizing the age variable, the significance increased in a chi-square test (*p* = 0.003), in which 21.4% of 45–54-year-olds did not receive the flyer and 29.8% of 45–54-year-olds did. The FIT return rate was higher in the flyer group (16.1%) than in the group not receiving the flyer (15.1%), but this difference was not statistically significant in the bivariate analysis. Of the 192 kits returned, twenty-seven participants (13 from the flyer group; 14 from the no-flyer group) were identified as having an abnormal result and were referred to their physician for further testing [21]. 

### 3.2. Predictors of Returning FIT Kits

A logistic model with flyer vs. no-flyer group predicting returning the FIT kit showed no difference by group in an unadjusted model (OR = 1.08, 95% CI: 0.79, 1.47) nor models adjusted by age and gender (OR = 1.21, 95% CI: 0.88, 1.66). The analysis found that female respondents in the older age categories had a higher probability of returning the FIT kits (Table 2). Compared to males, females were 1.78 times (95% CI: 1.19–2.64) as likely to return the FIT kit. The likelihood of returning FIT kits was higher among those aged 54–64 years (OR= 3.05, 95% CI: 1.83–5.10) and 65–74 years (OR = 5.03, 95% CI: 2.98–8.47) compared to those aged 45–54 years. No significant interaction between age and gender was identified.

## 4. Discussion

In this study, we evaluated the effect of a tailored educational flyer on the completion of CRC screening using a mailed FIT approach targeted at Midwestern rural residents in the USA. We created a simple, tailored, one-page flyer for rural men and women based on the feedback received from focus groups, which identified different barriers to CRC screening among men (e.g., fear or cost) and women (e.g., age). Contrary to our expectations, no difference was found in the FIT kit return rate between those that received the educational flyer and those that did not. We also found that older age (55–74, compared to 45–54) and being a female were significantly associated with a higher chance of returning FIT kits. 

Using small media, such as a flyer or brochure, has been known to be an effective intervention strategy for CRC screening, especially for stool-based tests [22]. However, more recent studies showed mixed evidence [23,24]. According to a systematic review study assessing the effect of educational interventions on CRC screening completion rates, 8 studies (of the 19 studies reviewed) found that the education intervention improved CRC screening, while 11 studies found that it did not significantly improve screening rates [24]. Most of these studies were conducted in urban settings. It is plausible that factors like the content, delivery, and intensity of education may impact effectiveness. For example, the content created for the educational intervention may not fit the context of the target population. In our study, the content of the flyer was based on focus group interviews with rural “agricultural” men and women and so may not have been persuasive for rural residents who are not in “agricultural” occupations. It may be also true that the age factor overrides the educational flyer effect. It might be that educational flyers specifically targeting younger rural residents are needed. Engaging primary care doctors in creating content would be essential. Also, the timing of mailing the educational flyer may affect the outcomes. In our study, participants received an educational flyer and the mailed FIT kits at the same time (e.g., flyer enclosed in the FIT kit). Other studies provided the educational flyer before or after the intervention, thus making the flyer serve as a prompt or reminder for participants [25,26]. Furthermore, other than a printed flyer format, alternative methods of delivering education (e.g., video/audio or other social media channels) might have been more effective in improving screening rates for this population [27]. For example, studies have shown that social media, such as Facebook, can be effective communication channels for promoting CRC screening for targeted age groups regardless of the geographic location [28,29]. 

Another plausible explanation for the lack of effectiveness of the educational flyer in increasing screening rates is that it addressed only the behavioral intention to be screened (i.e., ‘I will do this FIT kit’) by providing information about the perceived benefits and barriers to screening. The flyer may not have led to action/behavior change (i.e., completing the FIT kit) because the intention to be screened was not reinforced and might have been forgotten amidst participants’ other daily activities or priorities. Although we sent a one-time reminder letter, this might not be sufficient “cues to action”, requiring multiple reminders [30,31]. More studies supported these rationales by reporting multi-component interventions (i.e., combining educational interventions with telephone reminders or patient navigation interventions) to increase CRC screening rates [32,33,34]. Future research should explore the content, timing, and mode of delivery of educational interventions, as well as other multi-component strategies to increase cues to action (e.g., multiple reminders) and decrease perceived barriers (e.g., patient navigation) for rural residents. 

In our sample, the participants’ genders and ages were significantly associated with FIT return rates. In particular, older participants aged 55–64 and 65–74 were 3.05 and 5.03 times more likely to return the FIT kits, respectively, compared to those who were younger (45–54 years). This finding is consistent with the reported patterns of screening from the existing studies [8,9,35,36]. Potential reasons for low screening rates of younger-aged individuals include (1) incorrect knowledge about the recommended age for CRC screening and lower susceptibility, (2) perceived higher cost barriers (e.g., deductibles, transportation, or reduced workdays), and (3) less exposure to clinics or physicians who could recommend CRC screenings [36,37]. Future research may consider using tailored educational content focused on the recommended starting age (45) for CRC screening. This could include statistics about CRC in younger adults and the creation of social media campaigns featuring stories of public figures diagnosed with CRC at a younger age on a platform such as Twitter and Facebook [29,38].

The role of gender (female vs. male) in CRC screening completion is unclear. In our sample, females were 1.78 times more likely to return the kits than males, consistent with the national study [35]. Plausible reasons may include a tendency for women to be more likely to respond to surveys in general [39], higher perceived risk of CRC, and higher trust in medicine than men [40]. More discussions regarding the role of gender and age on FIT completion rates can be found in another study by the same authors [21]. 

Our study has several strengths. We assessed the effect of a one-page educational flyer tailored to rural men and women in the USA with a higher need for CRC screening on the FIT return rate. The findings from this randomized trial add more evidence to the existing literature regarding the effect of an educational flyer on CRC screening intervention by public health departments. 

This study has some limitations, including a lack of diverse ethnic groups, as the sample was primarily from the central USA, where 80% of the populations were non-Hispanic White Americans. The results may only be generalizable to other similar rural-based populations. Males may be under-represented in this sample, as one of the databases was specifically designed for breast and cervical cancer screening in women. Additionally, this study did not include information on other potential predictors of screening behaviors, including income, insurance, health literacy, or family history of cancer. The lack of consideration of these variables may weaken the external validity of the results. Although our intervention had a limited practical impact on improving screening adherence due to its non-significant results, reporting this study is important for two reasons. (1) It may help public health agencies make informed decisions regarding their CRC screening interventions given the competing priorities for allocating their limited resources, and (2) reporting all findings from well-designed studies, whether significant or not, is essential for effective dissemination and to reduce publication bias. Failing to report negative results can introduce bias into meta-analyses and result in wasted resources as others attempt to replicate earlier studies [41]. 

## 5. Conclusions

In conclusion, our study revealed that using a tailored educational flyer in the mailed FIT intervention does not make a meaningful difference in increasing FIT return rates for our sample of rural populations. Future studies need to consider the content, timing, and delivery methods of the educational intervention, as well as other strategies (e.g., multiple reminders) that can be combined to improve CRC screening adherence. This study also provides useful information for policy makers and program implementers regarding the potential roles of age and gender on the mailed FIT program and the future strategies to address the non-adherence of these populations. 

## Figures and Tables

**Table 1:** Demographic differences in those randomized to educational flyer intervention group and standard information group, July 2022 to December 2022.

Characteristic	No-Flyer Group (N = 622) N (%)	Flyer Group (N = 608) N (%)	Chi-Square (*p*-Value)
Gender			
Male	142 (22.8)	149 (24.5)	0.48 (0.49)
Female	480 (77.2)	459 (75.5)	
Age group			
45–54	133 (21.4)	181 (29.8)	11.5 (0.003)
55–64	279 (44.9)	248 (40.8)	
65–74	210 (33.7)	179 (29.4)	
Race/Ethnicity			
Non-Hispanic White	514 (84.4)	487 (82.7)	0.70 (0.70)
Non-Hispanic Other ^1^	19 (3.12)	19 (3.23)	
Hispanic	76 (12.5)	83 (14.1)	
Data Source			
EWMP	245 (39.4)	247 (40.6)	0.20 (0.66)
NCCR	377 (60.6)	361 (59.4)	
Zone			
1	416 (68.2)	413 (69.3)	0.48 (0.79)
12	121 (19.8)	109 (18.3)	
13	73 (12.0)	74 (12.4)	
FIT kit return status			
Returned	94 (15.1)	98 (16.1)	0.24 (0.63)
Not returned	528 (84.9)	510 (83.9)	

^1^ Non-Hispanic Other group include Asians (n = 8), Pacific Islanders/Native Americans (n = 22), and unknown (n = 23).

**Table 2:** Odds ratios and 95% confidence intervals from a logistic regression model for flyer vs. no-flyer group returning the FIT kit in a sample of 1230 individuals who were sent a FIT kit.

Predictor	Odds Ratio (95% CI ^1^)
Group	
Flyer	1.21 (0.88, 1.66)
No Flyer	Reference
Gender	
Male	Reference
Female	1.78 (1.19, 2.64)
Age group	
45–54	Reference
55–64	3.05 (1.83, 5.10)
65–74	5.03 (2.98, 8.47)

^1^ CI: Confidence Interval.

## Data Availability

Restrictions apply to the availability of these data. Data were obtained from the Nebraska Department of Health and Human Services (DHHS).
